# The Waiting Time of Prostate Cancer Patients in Poland

**DOI:** 10.3390/ijerph16030342

**Published:** 2019-01-26

**Authors:** Karolina Osowiecka, Sergiusz Nawrocki, Marcin Kurowicki, Monika Rucinska

**Affiliations:** 1 Department of Public Health, Collegium Medicum, University of Warmia and Mazury in Olsztyn, 10-082 Olsztyn, Al. Warszawska 30, Poland; k.osowiecka86@gmail.com; 2 Department of Public Health, Medical University of Warsaw, 02-097 Warszawa, Ul. Nielubowicza 5, Poland; 3 Radiotherapy Center Nu-Med, 82-300 Elblag, Ul. Królewiecka 146, Poland; sergiusz.nawrocki@me.com (S.N.); marcin.kurowicki@nu-med.pl (M.K.); 4 Department of Oncology and Radiotherapy, Medical University of Silesia in Katowice, 40-515 Katowice, Ul. Ceglana 35, Poland; 5 Department of Oncology, Collegium Medicum, School of Medicine, University of Warmia and Mazury in Olsztyn, 10-228 Olsztyn, Al. Wojska Polskiego 37, Poland

**Keywords:** prostate cancer, waiting time, health services

## Abstract

*Background*: Prostate cancer is the second most common reason of mortality due to cancer among men in Poland. The study aimed to determine the waiting time for diagnosis and treatment of prostate cancer. *Methods*: The study was carried out on patients treated for prostate cancer from May 2014 to February 2015 at five oncological centres in Poland. The median waiting time was measured from the time cancer was suspected to the histopathological diagnosis (SDI), from the cancer suspicion to the start of treatment (STI) and from the diagnosis to the start of treatment (DTI). *Results*: 123 males treated for prostate cancer were included for analysis. The median time for SDI, STI and DTI was 7.7, 18.7 and 8.7 weeks, respectively. Place of residence was the only factor which influenced STI (*p* = 0.003). For patients, who started treatment with radiation therapy DTI was longer than for other patients (*p* < 0.001). *Conclusions*: Median times of STI, SDI and DTI for prostate cancer patients in Poland are similar to the intervals described in other countries. Patients, who lived further from an oncology centre waited longer for treatment. The impact of waiting time in the case of prostate cancer on improving the prognosis is still unclear.

## 1. Introduction

Cancer is a significant global health care problem. At present, it is the dominant cause of deaths in the Polish population after cardiovascular diseases [1]. Prostate cancer is the second most common reason of mortality due to cancer among men in Poland, responsible for 8.8% of all cancer deaths [2]. Although the number of deaths is similar to those in Europe, relative 5-year survival in Poland is only 66.6% (about 1/5 less than the European average of 83.4%) [3,4].

Numerous studies have proved that there is a correlation between the extended time for diagnosis and the start of treatment, with worse outcomes of oncologic therapy of head and neck, breast, lung and cervix cancer [5,6,7,8,9,10,11]. In the case of prostate cancer such an association isn’t that clear.

The rationale for this study was to figure out if there are factors which may correlate with SDI/STI (SDI: suspicion—diagnosis interval; STI: suspicion—treatment interval) and could be responsible for prolonged waiting times for oncological treatment in Poland in general, not only for prostate cancer. We have previously published a similar study of the general Polish cancer patient population which included common cancer diagnoses [12]. We have deliberately excluded prostate cancer patients from the previous analyses due to the relatively slow progression of prostate cancer in relation to other common cancers which does not justify efforts to shorten STI. STI in the whole group of other than prostate cancer patients was 10.6 weeks which is significantly shorter than in prostate cancer cohort—18.7 weeks. We think that one of the reasons of inferior 5-year survival rates in cancer patients in Poland compared to EU (European Union) averages in general and in prostate cancer in particular is advanced stage at the beginning of the treatment and possibly also delayed treatment start. We feel that the factors associated with the delayed STI in prostate cancer in Poland (in our study) may be responsible for delays in other cancer diagnoses where the relation between STI and outcomes is evident and was reported in the literature.

## 2. Materials and Methods

The study was carried out on a group of 123 males treated for prostate cancer from 22 May 2014 to 19 February 2015 at five oncological centres in Poland. Eleven cancer centres in Poland varying in size, organisation, geographical location and financing services were invited to participate in this research. Five cancer centers decided to take part in the study (three public, regional provincial oncological centers where treatment was reimbursed with public resources and two private centers: in one, treatment was reimbursed with public resources, in another, treatment expenses were not reimbursed but covered by the owners and not by the patients). The sponsor of the study was Fundacja Onkologia 2025.

A questionnaire prepared especially for this study was validated in a group of 50 patients. Each patient was interviewed individually and the data obtained was supplemented with medical records and hospital databases.

The study protocol was approved by the Local Ethics Committee of the University of Warmia and Mazury, in Olsztyn, Poland (45/2014). All of the participants had submitted a signed consent form (informed consent attached).

Twenty-two patients with incomplete data of histopathology designation (missing exact data on histopathological report; in some cases of cancer progression there were no new histopathological examinations) were excluded from the analysis of the waiting time between the suspicion and the diagnosis, and between the diagnosis and start of the treatment.

The median, first quartile (Q1), and the third quartile (Q3) of the waiting time distributions were estimated. The median of waiting time was measured from the cancer suspicion to the diagnosis (SDI: suspicion—diagnosis interval), from the cancer suspicion to the start of treatment (STI: suspicion—treatment interval) and from the diagnosis to the start of treatment (DTI: diagnosis—treatment interval).

Cancer suspicion was defined as the date of the first visit to a doctor, with urinary disorders; or the date of PSA test in the case of patients without symptoms; or the date of the control visit when the recurrence of a previously treated cancer was observed. Therefore we included in our study both patients with primary prostate cancer diagnosis and with progression diagnosed during follow-up after initial radical for palliative treatment (PSA-based failure or dissemination to bones based on imaging). The diagnosis was defined as the date of obtaining histopathological results. The start of treatment was defined as the date of the initial prostate cancer treatment: surgery, radiotherapy (time of the first fraction), hormonal therapy or chemotherapy.

### Statistical Analysis

The validation of the questionnaire was carried out using Cohen’s Kappa. The waiting time distributions were compared with the theoretical normal distribution using the Shapiro–Wilk test. The differences in the waiting time between the subgroups were analysed with either the Mann–Whitney (for 2 subgroups) or the Kruskal–Wallis test, and the Dunn’s test post hoc (for >2 subgroups). Correlation between the waiting time and age was analysed using the Spearman correlation coefficient. A *p*-value of <0.05 was considered to be significant. The analysis was conducted using STATISTICA software (version 12.5) (StatSoft, Krakow, Poland) and SPSS Statistics 23.0 (IBM, Armonk, NY, USA).

## 3. Results

One hundred twenty-three consecutive patients were included in the analysis of the waiting time from the cancer suspicion to the start of the treatment. The analyses of the waiting time from cancer suspicion to the histopathological cancer confirmation and from the histopathological diagnosis to the beginning of the treatment were carried out on 101 patients. The median age of the responders was 65 years. The majority of the patients were pensioner (76%), married (84%). The first visit to a doctor for more than half of patients was due to urinary disorders (62%) (Table 1).

The median of the waiting time between cancer suspicion and the beginning of the treatment was 18.7 weeks (10.6–26.9 weeks, 25–75% IQR) (IQR: interquartile range). Among from analysed variables (education, martial status, place of residence, age, professional activity, presence of symptoms, first treatment choice, treatment intention and private or public service), only the place of residence was a factor which influenced the waiting time (*p* = 0.003). Patients, living in the biggest cities, waited the shortest time for start of the treatment, whereas the longest STI time was observed in case medium sized cities. The significant relationship was noted between patients residing in cities of 101,000–500,000 and cities of 50,000–100,000 (*p* = 0.004) and between cities of 50,000–100,000 and cities of less than 50,000 inhabitants (*p* = 0.009).

**Figure 1:**
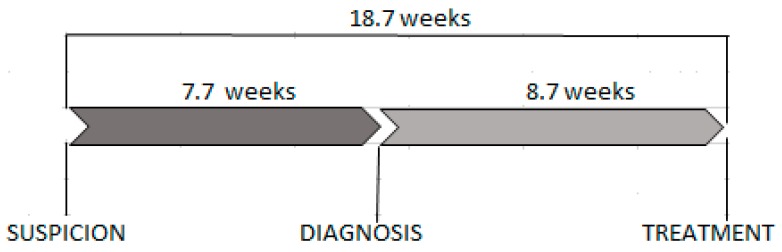
The waiting time for diagnosis and treatment.

There was no difference between small cities and villages observed. The median of waiting time since cancer suspicion till histopathological confirmation of cancer was 7.7 weeks (4.0–16.1 weeks, 25–75% IQR) and was not dependent on any analysed predictor. The median of waiting time since cancer diagnosis till the start of treatment was 8.7 weeks (4.6–14.1 weeks, 25–75% IQR). Patients, who began treatment with radiation therapy were waiting significantly longer than other patients (*p* < 0.001) (Table 2, Figure 1).

## 4. Discussion

The diagnosis of cancer affects patients with a lot of negative emotions and anxiety [13]. There is a widespread belief that this disease can quickly lead to death. Therefore, the diagnosis of cancer mobilises patients and doctors to make efforts to diagnose and start treatment as soon as possible.

The above analysis dedicated to prostate cancer is a continuation of the work assessing the time of diagnosis and the time to start treatment in other cancer locations [12]. The median of time from cancer suspicion to starting treatment (STI) in our report was 18.7 weeks. Similar time (18.6 weeks)—was observed in the Hansen et al. study in the Danish population [14]. In the remaining reviews found, this interval was much longer. In the Netherlands, the time from suspicion to starting any treatment was 33.9 weeks [15]. Stevens et al. noted that the median of waiting time from suspicion to radical radiotherapy of Canadian prostate cancer patients was 35.3 weeks [16]. Moreover, in men waiting for prostate cancer surgery in the UK the median of suspected time to treatment amounts 34.9 weeks [17].

In our previously published analysis, we indicated significant differences between the waiting time between cancer suspicion and treatment, cancer suspicion and diagnosis, diagnosis and treatment depends on different cancer localization [12]. The present study showed that in Poland the waiting time since prostate cancer suspicion till treatment compared to other oncological diseases described in our previously report was about eight weeks longer (10.6 weeks for all cancer patients; e.g., 10.9 weeks for breast cancer patients, 12.3 weeks for lung cancer patients, 9.1 weeks for colon cancer patients). A similar tendency was observed in the Dutch study where the difference in the median of STI of prostate cancer patients was as much as 29.3 weeks longer as compared to STI of breast cancer patients, while in the Danish analysis, only 4.5 weeks longer than median waiting time for all cancer patients [14,15].

In the Polish population surveyed, prostate cancer patients waited less for treatment in large cities, which are equipped in complex oncological centres. Also patients from small cities and villages are usually directed straight to centralised oncological centres and this could be the reason for the shorter waiting time in comparison with medium sized cities, where there could be some cancer facilities but usually monoprofiled and those facilities may delay patients journey to complex cancer centres. Unlike diagnosis-treatment interval, there was only a trend towards difference of waiting time for a specific therapy. Neither did we notice any significant difference in STI between groups depending on education, employment, marital status, use of private/public health services or the presence of disease symptoms. Similarly, in present paper age did not affect the acceleration of STI, while in some other studies, the time from suspicion to treatment was significantly longer in the elderly [16,18].

The median waiting time from cancer suspicion till diagnosis with histopathological confirmation (SDI) was similar to that in a Canadian review (7.7 and 7.6 weeks, respectively) [16]. A longer time to diagnosis was recorded in Australia, where the median was 9.3 weeks and in The Netherlands 19.6 weeks [15,19]. Comparing the results of this analysis with our previous study as in the Dutch paper, the time to diagnosis was the longest in the group of men with prostate cancer [12,15].

In our SDI analysis, none of the investigated factors exceeded the threshold of statistical significance. In the Baade et al. study [19], the factors that influenced the acceleration of the diagnosis were private insurance and, what’s interesting, the lack of clinical symptoms. Similarly, in UK the status of employment and the level of education had no impact [19].

In our report, the median of the time from cancer diagnosis to treatment (DTI) was 8.7 weeks. Comparable results for prostate cancer patient were obtained in UK (9.3 weeks) and in Netherland (7.9 weeks) [15,19]. Correspondingly, to STI and SDI, the median of DTI was dependent on the location of the neoplastic disease, e.g., for a patient diagnosed with prostate cancer, the waiting time for treatment from pathological diagnosis was almost three times longer than in the case of breast cancer patients and 2.3-fold longer for other cancer patients in Poland [12,15].

We showed that the only factor affecting the DTI time is the type of therapy chosen. An analogous observation was noted in Australia, where apart from the type of therapy, also the age, private insurance, retirement, distance from the centre influenced the time of starting the treatment [19].

Comparing the waiting times from histopathological diagnosis to a specific type of therapy (prostatectomy, hormonotherapy, chemotherapy or radiotherapy), awaiting for radiotherapy is the longest. For Polish men, the median of the waiting time for radiotherapy was 12.7 weeks, twice as high as in other locations previously published in our analysis and four weeks longer than waiting for surgery [12]. In Canada the waiting time for radiotherapy was 18.1 weeks, in Australia 17.3 weeks and was longer than waiting for a radical surgery by 6.3 weeks [16,19]. This dependence probably results from a much smaller number of radiotherapy centres compared to prostate cancer surgery units. The time of starting radiotherapy was counted from first fraction, and planning radiation therapy is complicated, involves different procedures and takes time.

The time expected for radical surgery in Poland, according to our paper, was about 8.6 weeks and was shorter than the described by Baade et al (11 weeks) and Redaniel et al. (13.6 weeks) [18,19]. Interestingly, the waiting time for surgical treatment was almost twice as high as in other cancer locations (8.6 vs. 4.1 weeks) [12]. In an Australian study, the waiting time for hormone therapy was 2.1 weeks [19]. The time of waiting for hormone therapy in our study was longer—6.3 weeks.

The STI and other time intervals cannot be compared directly between cited reports from other countries since inclusion criteria and other factors differed significantly. STI in the Canadian publication refers only to patients diagnosed with primary prostate cancer treated with teleradiotherapy in a small cohort of 41 patients [16]. In our cohort we have also patients treated with surgery and palliative patients treated for recurrence or metastases. In our cohort STI for patients treated with radiotherapy (median 21.3 weeks) was also much longer than treated with surgery (13.6 months). The Canadian authors stated that a large component of long wait times for radiotherapy in the Canadian cohort were patient driven. In the Dutch publication [15] primary prostate cancer cohort included 237 patients presented and referred from primary care services. In The Netherlands the median STI for prostate cancer was much longer than for other common cancers. For Poland we also observed analogical difference between prostate and other common cancers but of much smaller magnitude. SDI in Canada and in The Netherlands is comparable in Poland whereas STI is shorter in Poland for different patient populations (in Poland not only primary cancers but also recurrences treated with palliative hormonal treatment, chemotherapy or palliative radiotherapy where shorter times from diagnosis to start of treatment are obvious).

STI, SDI and DTI for prostate cancer from our analysis are longer than the targets set by the Polish National Program of Diagnosis and Treatment of Oncological Diseases. This program establishes that the time from cancer suspicion to diagnosis should not exceed four weeks (in our study this interval lasts 7.7 weeks) and from the diagnosis to the start of treatment should not exceed five weeks (in our study this time was 8.7 weeks). We have also shown that the factor that extends the time to treatment is the place of residence—faster in larger towns—which is related to the distance to cancer centres, as well as the type of therapy—the patients for radiotherapy awaited the longest.

In most cancers, the time from suspicion of neoplastic disease to the initiation of treatment is of great importance due to the dynamic progression of tumours. Prostate cancer is, however, characterised by entirely different biology and epidemiology. Its doubling time is up to 1100 days, in comparison, e.g., to breast cancer, where this time is 82 days [20]. Thanks to the use of methods such as DRE or PSA level, the number of newly diagnosed cases is increasing and relatively often prostate cancers are detected in early stages but their quicker detection did not translate into prostate cancer specific survival and overall survival [21,22]. The risk of progression of early prostate cancer is about 6 per cent in 10 year. That is why many men simply do not benefit from early treatment and must face the consequences of selected therapies [23]. Active surveillance is, therefore, a solution for some patients.

## 5. Conclusions

The time factor in prostate cancer is much less critical than in other cancerous diseases. Shortening the waiting time for diagnosis and treatment seems to reduce the stress associated with cancer, but its impact on improving the prognosis is controversial. It is noted that the distance between place of residence of patients and oncological center was related with waiting time prolongation.

## Figures and Tables

**Table 1:** Characteristics of the patients included in the analysis.

Characteristic	Patients Included in		Patients Included in	
	Suspicion-Treatment Analysis		Suspicion-Diagnosis, Diagnosis-Treatment Analysis	
	*n*	%	*n*	%
All patients	123	100	101	100
Age (years)	median 65; range 53–87			
Education				
Primary	28	23	24	24
Secondary	65	53	52	51
Higher	30	24	25	25
Place of residence				
City 101,000–500,000	40	33	32	32
City 50,000–100,000	17	14	14	14
City <50,0000	33	27	28	28
Village	33	27	27	27
Professional activity				
Active	26	21	24	24
Unemployed	3	2	2	2
Pensioner	94	76	75	74
Marital status				
Married	103	84	84	83
Single	10	8	9	9
Widower	10	8	8	8
Type of “patient route” starting points				
Symptoms	76	62	66	65
Prevention	38	31	32	32
Follow-up	9	7	3	3
Method of treatment beginning				
Surgery	26	21	14	14
Radiotherapy	44	36	35	35
Chemotherapy	3	2	3	3
Hormonal therapy	50	41	49	49
Treatment intention				
Curative	109	89	94	93
Palliative	14	11	7	7
Private medical services				
Yes	33	27	30	30
No	70	57	60	59
No data	20	16	11	11

**Table 2:** The impact of analysed factors on waiting time of prostate cancer patients.

Variable Name	Suspicion-Treatment Time (weeks) *n* = 123			Suspicion-Diagnosis Time (Weeks) *n* = 101			Diagnosis-Treatment Time (Weeks) *n* = 101		
	Median	(25–75% IQR)	*p-*Value	Median	(25–75% IQR)	*p-*Value	Median	(25–75% IQR)	*p-*Value
All patients	18.7	(10.6–26.9)		7.7	(4–16.1)		8.7	(4.6–14.1)	
Age			>0.05			>0.05			>0.05
Education									
Primary	18.1	(11.8–26.0)	0.86	8.4	(4.7–14.4)	0.70	8.0	(3.9–12.7)	0.55
Secondary	19.9	(9.7–27.3)		7.0	(3.4–17.5)		9.5	(5.9–15.9)	
Higher	15.8	(10.7–25.6)		8.7	(4.3–12.3)		7.3	(4.1–13.0)	
Place of residence									
City 101,000–500,000	14.9	(7.7–24.7)	0.003	8.7	(4.1–11.3)	0.11	7.3	(3.8–12.9)	0.34
City 50,000–100,000	25.0	(20.1–47.4)		11.5	(5.3–42.9)		13.4	(6.3–16.7)	
City <50,000	16.4	(9.1–23.3)		6.1	(3.0–9.4)		7.5	(4.7–15.1)	
Village	19.9	(13.1–26.9)		9.6	(4.0–21.0)		9.3	(4.6–12.9)	
Professional activity									
Active	17.3	(10.4–23.9)	0.09	4.4	(2.9–10.6)	0.20	9.9	(6.0–14.5)	0.63
Unemployed	36.9	(25.6–58.9)		17.9	(6.4–29.4)		13.3	(7.4–19.1)	
Pensioner	18.1	(10.6–26.9)		8.7	(4.1–17.4)		8.4	(4.0–14.1)	
Marital status									
Married	17.1	(9.7–26.1)	0.12	7.5	(3.9–17.4)	0.76	7.9	(4.1–13.8)	0.28
Single	22.9	(21.4–40.3)		9.0	(4.4–12.7)		12.3	(10.6–15.1)	
Widower	19.6	(15.4–24.3)		7.9	(6.3–12.6)		13.0	(5.9–15.0)	
Type of “patient route” starting points									
Symptoms	21.0	(11.7–26.8)	0.36	8.9	(4.3–17.4)	0.34	8.8	(3.6–14.6)	0.95
Prevention	14.8	(9.7–26.9)		5.9	(3.3–12.9)		8.3	(6.0–13.8)	
Follow-up	14.0	(13.6–20.1)		4.0	(4.0–32.3)		10.0	(2.0–16.1)	
Method of treatment beginning									
Surgery	13.6	(7.1–24.0)		8.3	(4.1–15.7)		8.6	(6.6–12.9)	
Radiotherapy	21.3	(14.6–27.7)	0.06	8.7	(4.7–16.1)	0.78	12.7	(8.7–17.7)	<0.001
Chemotherapy	34.7	(9.7–47.4)		32.0	(1.3–44.7)		2.7	(2.7–8.4)	
Hormonal therapy	17.2	(10.6–24.3)		6.6	(3.9–12.7)		6.3	(2.9–10.9)	
Treatment intention									
Curative	19.1	(10.6–26.1)	0.86	7.6	(4.0–16.1)	0.63	8.6	(4.9–14.1)	0.80
Palliative	14.5	(13.6–27.3)		9.7	(4.0–21.0)		10.0	(3.0–32.7)	
Private medical services									
Yes	19.9	(10.7–28.9)	0.69	4.6	(3.4–15.7)	0.15	8.5	(3.0–16.9)	0.79
No	21.0	(12.0–27.4)		9.2	(4.9–17.5)		8.6	(5.4–13.8)	

IQR: interquartile range.
